# Polymeric Multivalent Fc Binding Peptides‐Fabricated Clinical Compounding Bispecific Antibody Potentiates Dual Immunotherapy Targeting PD1 and CTLA‐4

**DOI:** 10.1002/advs.202408899

**Published:** 2024-11-28

**Authors:** Zongyu Liu, Hongyu Chu, Weidong Zhao, Chenguang Yang, Tongjun Liu, Na Shen, Zhaohui Tang

**Affiliations:** ^1^ Department of Colorectal and Anal Surgery The Second Hospital of Jilin University Changchun Jilin 130000 China; ^2^ Department of Gastrointestinal Colorectal and Anal Surgery China‐Japan Union Hospital of Jilin University Changchun Jilin 130033 China; ^3^ Key Laboratory of Polymer Ecomaterials Changchun Institute of Applied Chemistry Chinese Academy of Sciences Changchun Jilin 130022 China

**Keywords:** CTLA‐4, dual immunotherapy, Fc‐binding peptide, PD1, polymer

## Abstract

Dual Opdivo plus Yervoy immunotherapy, targeting the immune checkpoints PD1 and CTLA‐4, is successful in clinical use. However, it is associated with a high incidence of adverse events, and its therapeutic efficacy needs improving. In this study, polymeric multivalent Fc‐binding peptides (PLG‐Fc‐III‐4C) are employed to fabricate a bispecific antibody (PD1/CTLA‐4 BsAb) to potentiate dual immunotherapy targeting PD1 and CTLA‐4. The PD1/CTLA‐4 BsAb is prepared by mixing PLG‐Fc‐III‐4C with aPD1 and aCTLA‐4 in an aqueous solution for 3 h using the clinically optimal 3:1 proportion of aPD1 to aCTLA‐4. PD1/CTLA‐4 BsAb significantly inhibits tumors in MC38 colon cancer‐bearing mice more effectively than the combination of aPD1 and aCTLA‐4, with tumor suppression rates of 96.8% and 77.3%, respectively. It also induces a higher percentage of CD8^+^ T cells and increases the secretion of effector cytokines while reducing Treg levels in tumors compared to phosphate‐buffered saline, indicating significant tumor immunity regulation. Mechanistically, a 6.3‐fold increase in PD1/CTLA‐4 BsAb accumulation in tumors due to the tumor targeting ability of aPD1, and the PD1/CTLA‐4 BsAb significantly reduces the adverse colitis event in healthy mice, compared to aPD1 and aCTLA‐4. Thus, these findings provide a novel approach to enhance antitumor therapy using aPD1 and aCTLA‐4.

## Graphical abstract

This study investigates an approach to enhance dual immunotherapy targeting PD1 and CTLA‐4 by utilizing a clinically formulated bispecific antibody (PD1/CTLA‐4 BsAb) constructed with polymeric multivalent Fc‐binding peptides. The PD1/CTLA‐4 BsAb significantly improves tumor suppression, boosts CD8^+^ T cell immune responses, and reduces adverse events, offering a promising strategy for more effective cancer treatment.

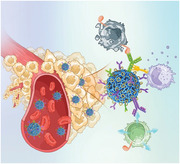

## 1 Introduction

Opdivo (nivolumab) plus Yervoy (ipilimumab) is dual immunotherapy targeting and blocking the immune checkpoints PD1 and CTLA‐4, and has been successfully applied in clinical cancer therapy,^[^ ^1^ ^]^ including colorectal cancer,^[^ ^2^ ^]^ hepatocellular carcinoma,^[^ ^3^ ^]^ and non‐small cell carcinoma.^[^ ^4^ ^]^ Less than 30% of cancer patients respond to immune checkpoint therapy, but treatment with Opdivo plus Yervoy at an optimum dosage ratio of 3:1 enhances antitumor efficacy. The overall 5‐year survival rate of patients with metastatic melanoma treated with Opdivo plus Yervoy is 52%, compared to 44% for Opdivo alone and 26% for Yervoy alone.^[^ ^5^ ^]^ The objective response rate of patients with microsatellite instability‐high/mismatch repair‐deficient metastatic colorectal cancer is 69%, with a 13% complete response rate.^[^ ^6^ ^]^ However, this efficacy is accompanied by frequent and serious immune‐related adverse events (IRAEs), necessitating a reduction in the recommended dose of ipilimumab.^[^ ^7^ ^]^ The incidence of IRAEs is 32.6% for Opdivo plus Yervoy, manifesting as abnormal liver function, pneumonia, hyponatremia, and gastrointestinal toxicity,^[^ ^8^ ^]^ compared to 12.8%^[^ ^9^ ^]^ for Opdivo alone. Therefore, a method that simultaneously enhances efficacy and reduces the adverse reactions of Opdivo plus Yervoy would greatly benefit cancer treatment.

Combined Opdivo plus Yervoy‐based cancer treatments involving various mechanisms, such as drugs or methods such as chemotherapy,^[^ ^10^, ^11^ ^]^ radiotherapy,^[^ ^12^ ^]^ chemoradiotherapy,^[^ ^13^ ^]^ tyrosine kinase inhibitors cabozantinib plus nivolumab and ipilimumab,^[^ ^14^ ^]^ histone deacetylases (HDAC) inhibitors,^[^ ^15^ ^]^ anti‐CD30 antibody‐conjugated monomethyl auristatin E,^[^ ^16^ ^]^ and live bacterial supplementation,^[^ ^17^ ^]^ have been successively developed. Although these therapies enhance tumor therapeutic efficacy, the majority induce more adverse effects than treatment with anti‐PD1 (aPD1) plus anti‐CTLA‐4 (aCTLA‐4) antibodies.

The symmetric tetravalent PD1/CTLA‐4 bispecific antibody cadonilimab (AK104), developed by Akeso, Inc. (China), with a PD1 scFv fragment and a CTLA‐4 scFv fragment ratio of 1:1,^[^ ^18^ ^]^ was recently approved for treating patients with recurrent or metastatic cervical cancer who have failed prior platinum‐based chemotherapy. The incidence of treatment‐related adverse events ≥ 3 grade is 27.0%.^[^ ^19^ ^]^ Additionally, AstraZeneca's monovalent PD1/CTLA‐4 bispecific antibody, MEDI5752, with a PD1 Fab and CTLA‐4 Fab ratio of 1:1,^[^ ^20^ ^]^ has entered clinical trials.^[^ ^21^ ^]^ However, these therapies do not maintain the optimal 3:1 aPD1 to aCTLA‐4 ratio found in Opdivo plus Yervoy therapy. Fabricating a bispecific antibody with this ratio using traditional biotechnology methods remains challenging and faces limitations such as complicated production processes, extensive screening, low yield, inconsistent quality, and significant impurities.^[^ ^22^ ^]^ Polymeric multivalent Fc‐binding peptides offer an opportunity to fabricate multi‐specific monoclonal antibodies into multi‐specific antibodies.^[^ ^23^ ^]^ This method preserves the activity of monoclonal antibodies without altering their native structure and overcomes the aforementioned limitations, potentially enabling the preparation of a PD1/CTLA‐4 bispecific antibody with the optimal 3:1 aPD1 to aCTLA‐4 ratio.

In this study, polymeric multivalent Fc‐binding peptides (Fc‐III‐4C) were conjugated with aPD1 and aCTLA‐4, resulting in the fabrication of a clinically compounding bispecific antibody. This approach aimed to enhance dual immunotherapy by simultaneously targeting PD1 and CTLA‐4. Specifically, multiple Fc‐III‐4C grafted poly(_L_‐glutamic acid) (PLG‐Fc‐III‐4C) was mixed with aPD1 and aCTLA‐4 at a 3:1 ratio in aqueous solution over 3 h to fabricate a PD1/CTLA‐4 bispecific antibody (PD1/CTLA‐4 BsAb). The physicochemical characterization and stability of the resulting PD1/CTLA‐4 BsAb were evaluated. The in vitro CD8^+^ T cell activation, in vivo tumor suppression, tumor immune regulation, biodistribution, and adverse effects of PD1/CTLA‐4 BsAb were assessed and compared to free mixed monoclonal antibodies (aPD1 + aCTLA‐4). The study findings provide new insight into improving cancer treatment using dual immune checkpoint inhibitors in clinical settings.

## 2 Results and Discussion

### 2.1 Preparation and Characterization of PLG‐Fc‐III‐4C and PD1/CTLA‐4 BsAb

**Figure 1:**
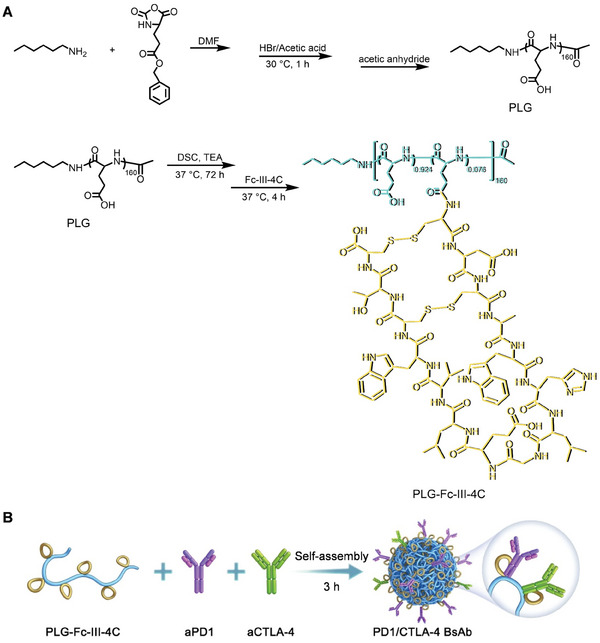
Schematic illustration of PD1/CTLA‐4 BsAb preparation. A) The synthesis route of PLG‐Fc‐III‐4C. B) PD1/CTLA‐4 BsAb preparation via the self‐assembly of PLG‐Fc‐III‐4C, aPD1, and aCTLA‐4, in which affinity exists between the Fc‐binding peptide Fc‐III‐4C and the monoclonal antibodies.

PLG was synthesized using the following steps. First, the ring‐opening polymerization of BLG‐NCA was initiated by *N*‐hexylamine. The ─NH_2_ end in the main chain of the polymer was then capped with acetic anhydride, followed by deprotection. PLG was used as the polymeric backbone and grafted with multiple Fc‐III‐4C units via a condensation reaction using *N, N*‐disuccinimidyl carbonate (DSC), and triethylamine (TEA) in *N, N*‐dimethylformamide (DMF) to obtain PLG‐Fc‐III‐4C (**Figure** **1**A). Subsequently, PLG‐Fc‐III‐4C, aPD1, and aCTLA‐4 self‐assembled to form bispecific antibodies (BsAb) in phosphate‐buffered saline (PBS) over 3 h on a shaker at 4 °C (Figure 1B).

**Figure 2:**
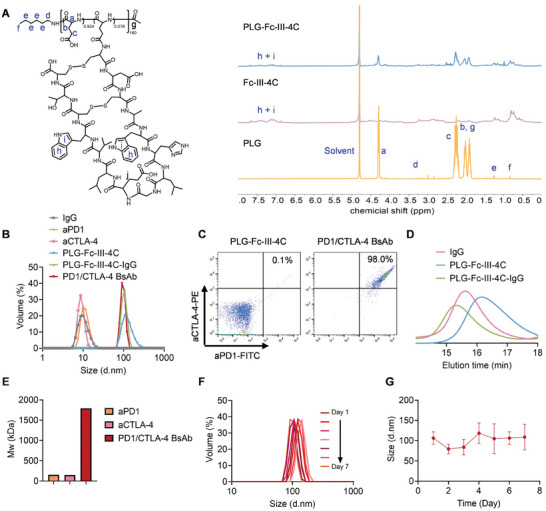
Characterization of PLG‐Fc‐III‐4C and PD1/CTLA‐4 BsAb. A) ^1^H NMR spectra of PLG, Fc‐III‐4C, and PLG‐Fc‐III‐4C. B) DLS was used to measure the hydrodynamic diameter of IgG, PLG‐Fc‐III‐4C, PLG‐Fc‐III‐4C‐IgG, and PD1/CTLA‐4 BsAb. C) Flow cytometric analysis of FITC‐labeled anti‐PD1 and PE‐labeled anti‐CTLA‐4 antibodies bound to BsAb. D) GPC comparison of molecular weights according to the elution curves of IgG, PLG‐Fc‐III‐4C, and PLG‐Fc‐III‐4C‐IgG. E) The molecular weight of aPD1, aCTLA‐4, and BsAb, as measured by asymmetric flow field‐flow fractionation coupled with multiangle laser light scattering. F) Storage stability of PLG‐Fc‐III‐4C‐IgG within 7 days detected by DLS when maintained at 4 °C. G) Quantitative data on the particle size of PLG‐Fc‐III‐4C‐IgG within 7 days, corresponding to Figure 2F (*n* = 3). The data are expressed as the mean ± SD.

PLG‐Fc‐III‐4C, signals at *δ* 4.20 ppm (a), *δ* 2.14 ppm (c), and *δ* 0.77 ppm (f) in the ^1^H NMR spectrum corresponded to the hydrogen on the α‐carbon atom, methylene on the side carboxyl group of PLG, and the *N*‐terminal methyl of PLG, respectively. Signals at *δ* 6.69‐7.73 ppm (h, i) were assigned to the hydrogen on the indole ring of Fc‐III‐4C. The degree of polymerization of PLG was determined to be 160. The integrated area ratio of h, i, and c in the ^1^H NMR spectrum and the number of structural hydrogens inferred that the grafting number of Fc‐III‐4C in PLG was 12.0 (**Figure** **2**A).

The dynamic light scattering (DLS) results showed particle sizes of 10.1, 10.0, 8.7, and 37.8 nm for IgG, aPD1, aCTLA‐4, and PLG‐Fc‐III‐4C, respectively. The sizes of PLG‐Fc‐III‐4C and PD1/CTLA‐4 BsAb were 106.0 and 91.3 nm, respectively, without volume distribution overlap in the particle size range of IgG or the monoclonal antibodies (mAbs) (Figure 2B). This indicated the successful preparation of PLG‐Fc‐III‐4C‐IgG and PD1/CTLA‐4 BsAb, with nearly all IgG or mAb bound to PLG‐Fc‐III‐4C, eliminating the need for separation. Transmission electron microscopy (TEM) images showed that the morphology of PLG‐Fc‐III‐4C was close to spherical, with a diameter of ≈50–60 nm (Figure S1, Supporting Information).

When PD1/CTLA‐4 BsAb was prepared with fluorescein‐isothiocyanate (FITC)‐labeled aPD1 and PE‐labeled aCTLA‐4, the flow cytometric analysis showed that 98.0% of the particles were FITC^+^PE^+^, compared to 0.1% in PLG‐Fc‐III‐4C without fluorescent antibodies (Figure 2C). Gel permeation chromatography (GPC) revealed that the retention time of PLG‐Fc‐III‐4C‐IgG was less than that of either PLG‐Fc‐III‐4C or IgG and was negatively correlated with molecular weights, indicating the successful binding of IgG to PLG‐Fc‐III‐4C (Figure 2D).

The average molecular weight (Mw) shown in Figure 2E and the input weight mass ratio of mAb and PLG‐Fc‐III‐4C (13:1) were used to conclude that the 1792 kDa PD1/CTLA‐4 BsAb consisted of 128 kDa of PLG‐Fc‐III‐4C and 1664 kDa of aPD1 + aCTLA‐4. This composition indicated that each BsAb contained 8.2 aPD1 and 2.7 aCTLA‐4. Additionally, PD1/CTLA‐4 BsAb remained stable in PBS at pH 7.4 for 7 days, with a consistent particle size ≈106 nm (Figure 2F,G). These results confirmed that the prepared PD1/CTLA‐4 BsAb was ready for use.

### 2.2 The Ability of BsAb to Activate CD8^+^ T Cells In Vitro

**Figure 3:**
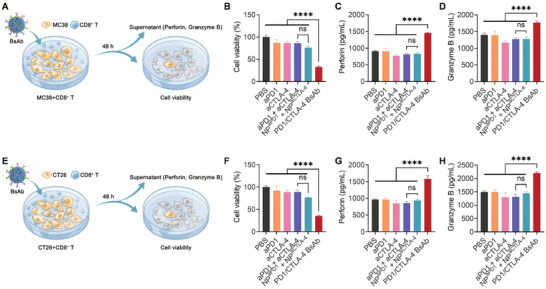
The in vitro effects of PD1/CTLA‐4 BsAb on CD8^+^ T cell activation and tumor cell killing ability. A–D) Schematic diagram of the process (A). Tumor cell viability detected using the CCK‐8 assay (B). ELISA analysis of perforin (C) and granzyme B (D) secreted by CD8^+^ T cells into the supernatant of co‐cultured MC38 cells and CD8^+^ T cells treated with PD1/CTLA‐4 BsAb. E–H) Schematic diagram of the process (E). Tumor cell viability detected using the CCK‐8 assay (F). ELISA analysis of perforin (G) and granzyme B (H) secreted by CD8^+^ T cells into the supernatant of co‐cultured CT26 cells and CD8^+^ T cells treated with PD1/CTLA‐4 BsAb. The data in (B–D) and (F–H) are expressed as the mean ± SD. One‐way ANOVA was used for statistical analyses between different groups; ns represents not significant, *p* > 0.05; **** *p* < 0.0001.

Two colon cancer cell lines, MC38 and CT26, were selected for co‐culture with CD8^+^ T cells, and the co‐cultured cells were treated with different drug formulations to study the ability of BsAb to activate T cells in vitro. The controls used in this and the following experiments were: PBS, non‐treated control; aPD1 and aCTLA‐4 single‐antibody controls; aPD1 + aCTLA‐4, simulated clinical dual immunotherapy; and NP^aPD1^ + NP^aCTLA‐4^ control with multi‐antigen binding valence comparable to that of BsAb. PD1 (alternative name CD279) and CTLA‐4 (alternative name CD152) are cluster molecules of differentiation. Multivalent ligands can bind to these receptor clusters, leading to a stronger binding avidity^[^ ^24^ ^]^ and a multivalent effect. Accordingly, PD1 or CTLA‐4 would be blocked more strongly when bound by multivalent inhibitors, and NP^aPD1^ + NP^aCTLA‐4^ was used to check the contribution of multivalence to the enhanced activation effect of aPD1 + aCTLA‐4. When MC38 cells were co‐cultured with CD8^+^ T cells under various treatments (**Figure** **3**A), aPD1 + aCTLA‐4 induced 14.1% MC38 cell death by CD8^+^ T cells. In contrast, PD1/CTLA‐4 BsAb caused 67.2% MC38 cell death, with a significant difference (Figure 3B). However, NP^aPD1^ + NP^aCTLA‐4^ caused 24.0% MC38 cell death compared to aPD1 + aCTLA‐4, without statistical significance, indicating a slight contribution of the multivalence effect to enhancing CD8^+^ T cell ability. Regarding CD8^+^ T cell activation, cytokine levels in the supernatant of the cultured cells were tested by enzyme‐linked immunosorbent assay (ELISA), including perforin and granzyme B, which mainly mediate the cytotoxic activity of CD8^+^ T cells against cancer.^[^ ^25^ ^]^ aPD1 + aCTLA‐4 and NP^aPD1^ + NP^aCTLA‐4^ resulted in a slight change compared to PBS. Only PD1/CTLA‐4 BsAb significantly increased perforin (1456.8 vs 908.5 pg mL^−1^, Figure 3C) and granzyme B (1774.3 vs 1401.5 pg mL^−1^, Figure 3D) concentrations, compared with PBS. Similar results were obtained when CT26 cells were co‐cultured with CD8^+^ T cells under various treatments (Figure 3E). aPD1 + aCTLA‐4 induced 10.9% CT26 cell death by CD8^+^ T cells. In contrast, PD1/CTLA‐4 BsAb caused 65.1% CT26 cell death, with a significant difference (Figure 3F). However, NP^aPD1^ + NP^aCTLA‐4^ caused 23.1% CT26 cell death, without statistical significance compared to that caused by aPD1 + aCTLA‐4, indicating the slight contribution of the multivalence effect to enhancing CD8^+^ T cell ability. Regarding the cytokine levels in the supernatant of the cultured cells, the concentration of perforin and granzyme B in cells treated with aPD1 + aCTLA‐4 and NP^aPD1^ + NP^aCTLA‐4^ was not increased than in cells treated with PBS. Only PD1/CTLA‐4 BsAb significantly increased perforin (1580.4 vs 965.5 pg mL^−1^, Figure 3G) and granzyme B (2205.9 vs 1490.4 pg mL^−1^, Figure 3H) concentrations compared with PBS. Therefore, these results indicate that BsAb outperformed mAbs in multiple aspects by activating CD8^+^ T cells and further killing tumor cells and that the multivalence effect had a slight contribution.

### 2.3 Tumor Suppression and Immunomodulation Capability of BsAb In Vivo

**Figure 4:**
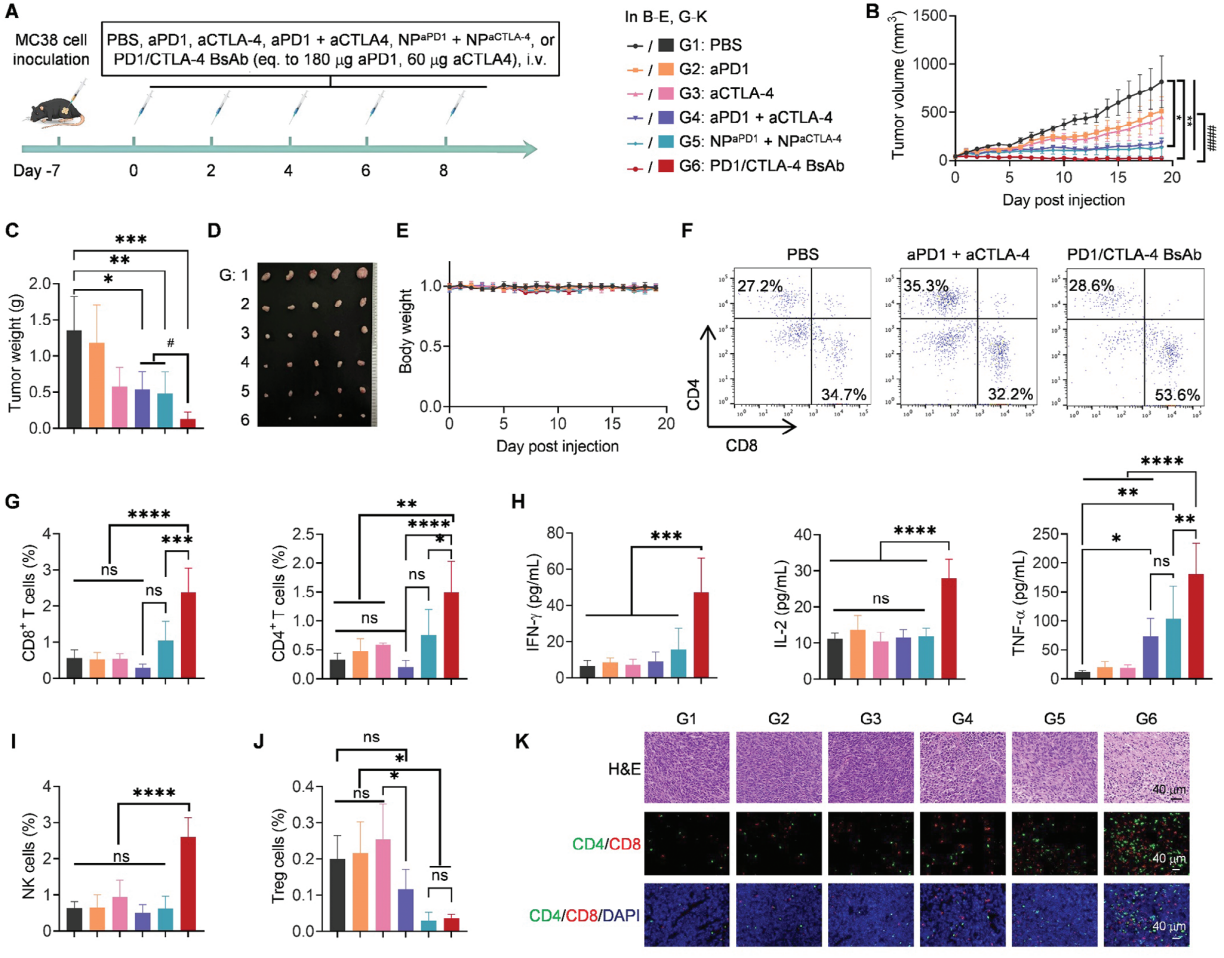
The in vivo tumor‐suppressing ability of PD1/CTLA‐4 BsAb and the underlying mechanism in MC38‐bearing C57BL/6 mice. A) Schedule for tumor cell inoculation and drug treatment of the mice. B) Tumor growth curves of mice in different treatment groups over 20 days. The data are expressed as the mean ± SEM. *p < 0.05, **p < 0.01 are analyzed by one‐way ANOVA; ^####^ *p* < 0.0001 analyzed by the unpaired student's t‐test. C) Tumor weights were collected on day 19. D) Photos of tumors collected on day 19. E) Changes in body weight of the mice. F,G) Representative plots (F) and quantitative analysis (G) of flow cytometry detecting the proportion of intratumoral CD8^+^ T (CD3^+^CD8^+^/total cells) and CD4^+^ T (CD3^+^CD4^+^/total cells) cells on day 19. The gating strategy is shown in Figure S4 (Supporting Information). H) Cytokine levels were detected by ELISA in the serum of tumor‐bearing mice on day 19 post‐different treatments, including IFN‐γ, IL‐2, and TNF‐α. I,J) The proportion of intratumoral NK cells (CD3^−^NK1.1^+^/total cells) and Treg cells (CD3^+^CD4^+^CD25^+^FOXP3^+^/total cells). The gating strategy is shown in Figure S4 (Supporting Information). K) Hematoxylin and eosin (H&E) staining of tumors and immunofluorescence staining of CD4^+^ and CD8^+^ T cells in the tumors. The tumor samples were separated from tumor‐bearing mice on day 19 post‐different treatments. Red, CD8; green, CD4; and blue, DAPI. Scale bars: 40 µm. The data in (C), (E), and (G–J) are expressed as the mean ± SD. ns, not significant; *p < 0.05, **p < 0.01 as analyzed by one‐way ANOVA. In C, ^#^ *p* < 0.05 as analyzed by the unpaired student's t‐test.

MC38‐bearing mice were subjected to different treatment, including PBS (G1), aPD1 (G2), aCTLA‐4 (G3), aPD1+aCTLA‐4 (G4), NP^aPD1^ +NP^aCTLA‐4^ (G5), and PD1/CTLA‐4 BsAb (G6), i.v. five times with 180 µg of aPD1 or/and 60 µg of aCTLA4 on days 0, 2, 4, 6, and 8 to determine the tumor‐suppressing ability of BsAb (**Figure** **4**A). The tumor growth curve demonstrated that single aPD1 and aCTLA‐4 treatments had weak inhibitory effects and relatively fast tumor growth, with a much lower tumor inhibition rate than free mixed aPD1 + aCTLA‐4 (Tumor suppression rate, TSR: 37.7%, 44.8% vs 77.3%; Figure 4B). NP^aPD1^ + NP^aCTLA‐4^ strongly inhibited tumor growth, and PD1/CTLA‐4 BsAb suppressed tumor growth the most (TSR: 83.6% vs 96.8%). In addition, significant differences were seen in tumor volumes between the PD1/CTLA‐4 BsAb group and all the other groups when analyzed using the unpaired student's t‐test. In contrast, no significant difference in tumor volumes was seen between the aPD1 + aCTLA‐4 and NP^aPD1^ + NP^aCTLA‐4^ groups. The tumor weights in G1–G6 were 1.35, 1.19, 0.57, 0.54, 0.48, and 0.13 g, respectively (Figure 4C). The tumor appearances were consistent with the tumor growth trends and weights (Figure 4D). These findings revealed the superior tumor‐inhibiting ability of PD1/CTLA‐4 BsAb, which is less reliant on multivalence effects, as the combined delivery of the two single antibodies does not significantly outperform the mixed monotherapies. As body weight loss reflects the systematic toxicity of drugs, the body weight changes were compared (Figure 4E). No body weight loss in any group exceeded 3%, and the losses were steadily maintained over the observation period. Flow cytometry and ELISA were used to evaluate the levels of immune cells and systemic cytokines in tumors after different treatments to understand the mechanism of PD1/CTLA‐4 BsAb in inhibiting tumors and activating immunity. The number of CD8^+^ and CD4^+^ T cells in the tumors of mice injected with PD1/CTLA‐4 BsAb significantly increased compared with PBS treatment (CD8^+^ T cells: 2.38% vs 0.56%; CD4^+^ T cells: 1.50% vs 0.33%) and were significantly higher than in the other groups (Figure 4F,G; Figure S2, Supporting Information). Since interferon (IFN)‐γ, interleukin (IL)‐2, and tumor necrosis factor (TNF)‐α are common cytokines secreted by activated T cells,^[^ ^26^ ^]^ they were examined to confirm the activation of elevated T cells induced by PD1/CTLA‐4 BsAb. The ELISA assay showed that serum IFN‐γ, IL‐2, and TNF‐α concentrations increased in mice post‐PD1/CTLA‐4 BsAb treatment compared with those treated with PBS (IFN‐γ: 47.3 vs 6.7 pg mL^−1^; IL‐2: 27.8 vs 11.2 pg mL^−1^; TNF‐α: 181.0 vs 11.9 pg mL^−1^). As aCTLA‐4 plays a role primarily in reducing the number of Treg cells induced by the antibody‐dependent cell‐mediated cytotoxicity (ADCC) effect in activating natural killer (NK) cells when its Fc fragment binds to their FcγR,^[^ ^27^ ^]^ levels of NK cells, IL‐12, central to NK effector function,^[^ ^28^ ^]^ and Treg cells were measured. The results indicated that PD1/CTLA‐4 BsAb significantly increased the proportion of NK cells (2.60% vs 0.63%, Figure 4I; Figure S2, Supporting Information) and IL‐12 levels (68.74 vs 30.50 pg mL^−1^, Figure S3, Supporting Information) in the tumors of mice while reducing the proportion of Treg cells (0.04% vs 0.20%, Figure 4J) compared to PBS. Finally, hematoxylin and eosin (H&E) staining and immunofluorescence staining of CD8 and CD4 expression were performed on the tumor tissue (Figure 4K). The H&E staining showed a large area of tumor cell death with nuclear dissolution and tissue cavities in the tumors in the PD1/CTLA‐4 BsAb group, indicating that the tumors were recognized and killed by the immune system. The remaining groups of tissues also showed varying degrees of tumor cell death post‐aPD1 + aCTLA‐4 and NP^aPD1^ + NP^aCTLA‐4^ treatments, with a trend consistent with the tumor growth curve. Immunofluorescence staining revealed a significant increase in CD8^+^ and CD4^+^ cells in the tumors of mice post‐PD1/CTLA‐4 BsAb treatment, with respective increases in red and green staining, verifying increased CD8^+^ and CD4^+^ T cell infiltration at the tumor site, and consistent with the flow cytometry results. Thus, these results illustrated the better tumor‐inhibiting ability of PD1/CTLA‐4 BsAb than that of free mixed aPD1 + aCTLA‐4.

**Figure 5:**
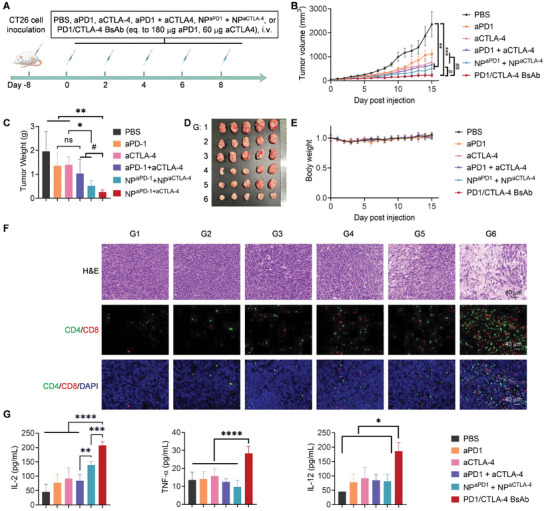
The in vivo tumor‐suppressing ability of PD1/CTLA‐4 BsAb and the underlying mechanism in CT26‐bearing C57BL/6 mice. A) Schedule for tumor cell inoculation and drug treatment of the mice. B) Tumor growth curves of treated mice in different groups over 16 days. The data are expressed as the mean ± SEM. **p < 0.01, ***p < 0.001 as analyzed by one‐way ANOVA; ^#^ *p* < 0.05, ^##^ *p* < 0.01 as analyzed by the unpaired student's t‐test. C) Tumor weights collected on day 15. D) Photos of tumors collected on day 15. E) Changes in mice body weights. F) Hematoxylin and eosin (H&E) staining of tumors and immunofluorescence staining of CD4^+^ and CD8^+^ T cells in the tumors. The tumor samples were separated from tumor‐bearing mice on day 15 post‐different treatments. Red, CD8; green, CD4; and blue, DAPI. G) Cytokine levels were detected by ELISA in the serum of tumor‐bearing mice on day 19 post‐different treatments, including IFN‐γ, TNF‐α, and IL‐12. In (C), (E), and (G), the data are expressed as the mean ± SD. *p < 0.05, **p < 0.01, ***p < 0.001, ****p < 0.0001 as analyzed by one‐way ANOVA. In (C), ^#^ *p* < 0.05 was analyzed by the unpaired student's t‐test. In (D) and (F), G1–6 represent PBS, aPD1, aCTLA‐4, aPD1 + aCTLA‐4, NP^aPD1^ + NP^aCTLA‐4^, and PD1/CTLA‐4 BsAb, respectively.

Next, another tumor model, the CD26 model was employed to assess the antitumor effect the PD1/CTLA‐4 BsAb (**Figure** **5**A). The tumor growth curve demonstrated that single aPD1 and aCTLA‐4 treatments were inhibitory to some extent, and aCTLA‐4 contributed more to the stronger tumor inhibition of free mixed aPD1 + aCTLA‐4 (TSR: 52.2%, 66.5% vs 71.8%; Figure 5B). NP^aPD1^ + NP^aCTLA‐4^ more strongly inhibited tumor growth, and PD1/CTLA‐4 BsAb most strongly suppressed tumor growth (TSR: 78.2% vs 90.3%). In addition, significant differences were seen in tumor volumes between the PD1/CTLA‐4 BsAb group and all other groups when analyzed using the unpaired student's t‐test or one‐way ANOVA. In contrast, no significant difference in tumor volumes was seen between the aPD1 + aCTLA‐4 and NP^aPD1^ + NP^aCTLA‐4^ groups. The tumor weights of mice in G1–G6 were 1.96, 1.36, 1.40, 1.03, 0.52, and 0.26 g, respectively (Figure 5C). The tumor appearances were consistent with the tumor growth trends and weights (Figure 5D). These findings revealed the superior tumor‐inhibiting ability of PD1/CTLA‐4 BsAb, which is less reliant on multivalence effects, as the combined delivery of the two single antibodies does not significantly outperform the mixed monotherapies. As body weight loss reflects the systematic toxicity of drugs, the body weight changes were compared (Figure 5E). No body weight changes were observed in mice in the treatment groups compared with the PBS group, which were maintained steadily over the observation period. H&E staining and immunofluorescence staining to detect CD8 and CD4 expression were performed on the tumor tissue (Figure 5F). The H&E staining showed that the tumors in the PD1/CTLA‐4 BsAb group had a large area of tumor cell death with nuclear dissolution and tissue cavities, indicating that the tumors were recognized and killed by the immune system. The remaining groups of tissues also showed varying degrees of tumor cell death post‐aPD1 + aCTLA‐4 group and NP^aPD1^ + NP^aCTLA‐4^ treatment, with a trend consistent with the tumor growth curve. Immunofluorescence staining revealed a significant increase in CD8^+^ and CD4^+^ cells in the tumors of mice post‐PD1/CTLA‐4 BsAb treatment, with respective increases in red and green staining, verifying increased CD8^+^ and CD4^+^ T cell infiltration at the tumor site, and consistent with the flow cytometry results. ELISA assays showed that serum IL‐2, TNF‐α, and IL‐12 concentrations increased in mice post‐PD1/CTLA‐4 BsAb treatment compared with those treated with PBS (IL‐2: 207.1 vs 45.7 pg mL^−1^; TNF‐α: 28.3 vs 13.5 pg mL^−1^; IL‐12: 185.0 vs 45.7 pg mL^−1^) (Figure 5G). Therefore, these results further confirmed the superior tumor‐inhibiting ability of PD1/CTLA‐4 BsAb than free mixed aPD1 + aCTLA‐4.

### 2.4 Tumor Targeting and Adverse Toxicity‐Reducing Ability of PD1/CTLA‐4 BsAb

**Figure 6:**
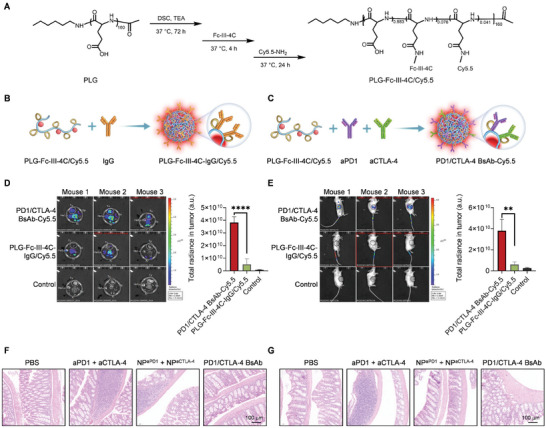
In vivo biological distribution and adverse toxicity of PD1/CTLA‐4 BsAb. A) Synthesis route of PLG‐Fc‐III‐4C/Cy5.5. B,C) Schematic illustration of PLG‐Fc‐III‐4C‐IgG/Cy5.5 (B) and PD1/CTLA‐4 BsAb‐Cy5.5 preparation (C). D) Fluorescence imaging of ex vivo tumors and major organs and the quantitative total radiance of the tumors in MC38‐bearing mice (*n* = 3) 24 h‐post PLG‐Fc‐III‐4C‐IgG/Cy5.5 or PD1/CTLA‐4 BsAb‐Cy5.5 treatment. He, Li, Sp, Lu, Ki, and Tu represent the heart, liver, spleen, lung, kidney, and tumor, respectively. The tumors are labeled with white circles in the pictures. E) Fluorescence imaging of in vivo tumors and major organs and the quantitative total radiance in the tumors of CT26‐bearing mice (*n* = 3) 24 h post‐PLG‐Fc‐III‐4C‐IgG/Cy5.5 or PD1/CTLA‐4 BsAb‐Cy5.5 treatment. Tumors are labeled with black circles in the pictures. F,G) H&E staining of Swiss‐rolled colons of healthy C57BL/6 mice (F) and healthy BALB/c mice (G) treated with PBS, aPD1 + aCTLA‐4, NP^aPD1^ + NP^aCTLA‐4^, or PD1/CTLA‐4 BsAb. Scale bars, 100 µm. In (D) and (E), the data are expressed as the mean ± SD. *p < 0.05, **p < 0.01 as analyzed by one‐way ANOVA.

PD1 is expressed by tumors, while CTLA‐4 is expressed more by blood CD4^+^ T cells,^[^ ^29^ ^]^ which may explain the stronger toxicity of aCTLA‐4 than aPD1. aPD1 in PD1/CTLA‐4 BsAb was hypothesized to induce a tumor‐targeting distribution. Accordingly, its tumor biodistribution was evaluated to further elucidate the mechanism underlying the superior role of PD1/CTLA‐4 BsAb in tumor inhibition and immune re‐invigoration. The fluorescent dye Cy5.5 was grafted onto the main ─COOH of PLG‐Fc‐III‐4C via one‐pot synthesis with PLG‐Fc‐III‐4C, in which Cy5.5‐NH_2_ was added after Fc‐III‐4C (**Figure** **6**A), and its loading content was calculated according to the data in Table S2 (Supporting Information). Then, PLG‐Fc‐III‐4C‐IgG/Cy5.5 or PD1/CTLA‐4 BsAb‐Cy5.5 was prepared by mixing IgG or aPD1 + aCTLA‐4 with PLG‐Fc‐III‐4C/Cy5.5 (Figure 6B,C). Then, the biodistribution of PLG‐Fc‐III‐4C‐IgG/Cy5.5 and PD1/CTLA‐4 BsAb‐Cy5.5 was evaluated in MC38‐bearing C57BL/6 mice and CT26‐bearing BALB/c mice. The fluorescence imaging of ex vivo organs in MC38‐bearing mice treated with PBS, PLG‐Fc‐III‐4C‐IgG/Cy5.5, and PD1/CTLA‐4 BsAb‐Cy5.5 showed enriched Cy5.5 signals in the tumors of mice treated with PD1/CTLA‐4 BsAb‐Cy5.5, with fluorescence intensity higher than those treated with PLG‐Fc‐III‐4C‐IgG/Cy5.5 (average radiance: 38.3 × 10^9^ vs 5.2 × 10^9^ a.u.). In addition, in vivo imaging for CT26‐bearing mice showed enriched Cy5.5 signals in the tumors of mice treated with PD1/CTLA‐4 BsAb‐Cy5.5, with fluorescence intensity higher than those treated with PLG‐Fc‐III‐4C‐IgG/Cy5.5 (average radiance: 38.0 × 10^9^ vs 6.1 × 10^9^ a.u.). These findings revealed that PD1/CTLA‐4 BsAb was enriched in the tumors, facilitating more aCTLA‐4 delivery to the tumor site than aPD1 + aCTLA‐4, which would benefit immune re‐invigoration and tumor inhibition.

Then, the adverse toxic effects of aPD1 + aCTLA‐4, NP^aPD1^ + NP^aCTLA‐4^, and PD1/CTLA‐4 BsAb were compared. Epithelial tissue, like that of the colon, is colonized by a diverse array of microorganisms and subsequently infiltrated by large populations of tissue‐resident T cells (Trms),^[^ ^30^ ^]^ which may be specific for microbial antigens and become reactivated following the blockade of CTLA‐4 and/or PD1 inhibitory receptors. Reactivated Trms show elevated cytotoxicity, proliferation, and inflammatory cytokine (IFN‐γ) programs. Myeloid cells respond to IFN‐γ and other cytokines by amplifying the inflammatory response and recruiting T cells from the circulation, thereby overwhelming Treg‐mediated suppression.^[^ ^31^ ^]^ Thus, colitis is among the most frequent and problematic immune‐mediated adverse events that are associated with dual checkpoint inhibition.^[^ ^32^ ^]^ Healthy mice without tumors were used to compare the incidence of colitis with immune cell infiltration after different treatments (Figure 6F,G). Healthy BALB/c or C57BL/6 mice were injected with PBS, aPD1 + aCTLA‐4, NP^aPD1^ + NP^aCTLA‐4^, or PD1/CTLA‐4 BsAb (eq. to 180 µg/mouse aPD1, 60 µg/mouse aCTLA‐4) through the tail vein every 2 days for a total of three doses. On day 5 post‐drug administration, the mice were euthanized, their intestines were collected, and H&E staining was used to observe the abundance of blue‐stained inflammatory immune cells in the basal layer of their intestines. Similar results were found in the two types of healthy mice. A large amount of lymphocyte infiltration and proliferation was found in the intestinal basal layer of mice in the aPD1 + aCTLA‐4 group, which was significantly different from the other groups, indicating that adverse reactions had been triggered. The NP^aPD1^ + NP^aCTLA‐4^ group showed a small amount of lymphocyte infiltration. No colitis was observed in the mice in the PD1/CTLA‐4 BsAb group, which was similar to the PBS group. The above results prove that PD1/CTLA‐4 BsAb can effectively reduce the toxic side effects of aPD1 + aCTLA‐4.

## 3 Conclusion

**Scheme 1:**
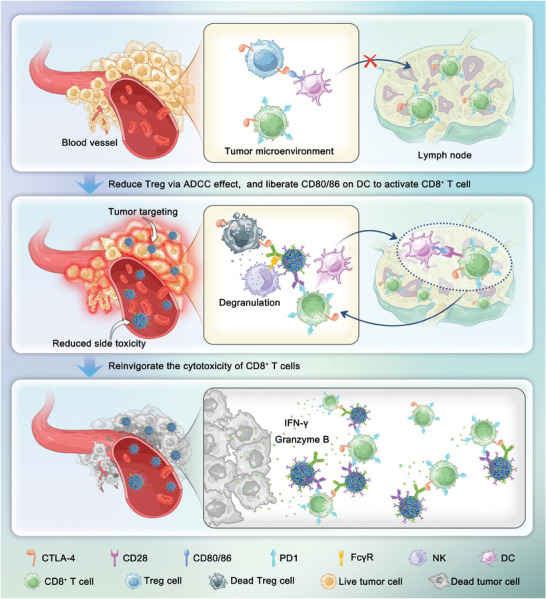
Schematic illustration for the possible mechanism underlying the PD1/CTLA‐4 BsAb‐induced tumor‐killing ability of CD8^+^ T cells.

In summary, this study developed a polymeric multivalent Fc‐binding peptide‐fabricated bispecific antibody (BsAb) for dual immunotherapy targeting PD1 and CTLA‐4. The BsAb was prepared by mixing poly(*L*‐glutamic acid) grafted with multiple Fc‐III‐4C (PLG‐Fc‐III‐4C) with aPD1 and aCTLA‐4 in an aqueous solution for 3 h, using the clinically optimal ratio of 3:1 aPD1 to aCTLA‐4. The PD1/CTLA‐4 BsAb significantly inhibited tumor growth more effectively than the combination of aPD1 and aCTLA‐4 in MC38‐bearing mice, with tumor suppression rates of 96.8% and 77.3%, respectively. It also induced a higher percentage of CD8^+^ T cells in tumors (2.38% vs 0.56%) and increased effector cytokine secretion (IFN‐γ: 47.3 vs 6.7 pg mL^−1^; IL‐2: 27.8 vs 11.2 pg mL^−1^; TNF‐α: 181.0 vs 11.9 pg mL^−1^) while reducing Treg levels (0.04% vs 0.20%) compared to PBS, significantly modulating tumor immunity. PD1/CTLA‐4 BsAb demonstrated significant tumor accumulation, with a 6.3‐fold increase compared to PLG‐Fc‐III‐4C‐IgG in MC38‐bearing mice, and significantly reduced the adverse colitis event related to toxicity observed with aPD1 + aCTLA‐4 in healthy mice (**Scheme** **1**). Thus, this study offers a potential and effective method to enhance antitumor therapy using aPD1 and aCTLA‐4.

## 4 Experimental Section

### Materials

γ‐Benzyl‐*L*‐glutamate‐*N*‐carboxyanhydride (BLG‐NCA) was purchased from Chengdu Enlai Biological Technology Co., Ltd., China, purified by recrystallization from ethyl acetate, and dried in vacuum at room temperature before use. *N, N*‐dimethylformamide (DMF) was dried over CaH_2_ for 72 h and distilled under reduced pressure. Fc‐III‐4C (amino acid sequence: NH_2_‐Cys‐Asp‐Cys‐Ala‐Trp‐His‐Leu‐Gly‐Glu‐Leu‐Val‐Trp‐Cys‐Thr‐Cys‐COOH; two disulfide bonds form in Cys1‐Cys15 and Cys2‐Cys13) was purchased from GL Biochem Ltd. (Shanghai, China). *N, N*‐disuccinimidyl carbonate (DSC) and triethylamine (TEA) were obtained from Sigma (Merck, Darmstadt, Germany) and Energy Chemical (Shanghai, China), respectively. Cy5.5‐NH_2_ was purchased from Wuhan Duofluor Inc. (Wuhan, China).

The in vivo injectable anti‐mouse PD1 antibody (aPD1, Rat IgG1, κ; catalog: BE0146) and anti‐mouse CTLA‐4 antibody (aCTLA‐4, Rat IgG1, κ; catalog: BE0164) were purchased from Bio X Cell (West Lebanon, NH, USA). Mouse IgG (catalog: SP031) was obtained from Solarbio Science & Technology (Beijing, China). Fetal bovine serum (FBS) was purchased from Zhejiang Tianhang Biotechnology Co., Ltd. (Zhejiang, China). Penicillin and streptomycin were purchased from Huabei Pharmaceutical (Shijiazhuang, China). ELISA kits were purchased from Servicebio Technology Co., Ltd. (Wuhan, China). All antibodies used for flow cytometry were purchased from BioLegend (San Diego, CA, USA.; Detailed information can be found in Table S1 (Supporting Information). The Easysep mouse CD8^+^ T cell isolation kit was purchased from Stemcell Technologies (Vancouver, Canada). The Cell Counting Kit‐8 (CCK‐8) was purchased from Dojindo Chemical Technology Co. Ltd. (Shanghai, China). All other reagents and solvents were purchased from Energy Chemical (Shanghai, China). Purified deionized water was prepared using a Milli‐Q plus system (Millipore Co., Billerica, MA, USA).

### Synthesis of poly (*L*‐Glutamic Acid)

PLG was synthesized via the ring‐opening polymerization of BLG‐NCA and subsequent deprotection. Briefly, BLG‐NCA (10.0 g, 38 mmol) was dissolved in 70 mL of anhydrous DMF under a nitrogenous atmosphere. Then, 1‐hexylamine (24.0 mg, 0.2375 mmol) solubilized in anhydrous DMF (4.7 mL) was added. Polymerization was performed at 30 °C for 3 days. Then, acetic anhydride (1.21 g, 11.86 mmol) was added to further react for 24 h at 30 °C. The reaction mixture was precipitated using an excess of ether to obtain a white solid poly (γ‐benzyl *L*‐glutamate) (PBLG). PBLG was then dissolved in 77 g of trifluoroacetic acid, and 21 g of HBr/acetic acid (33 wt.%) was added. The solution was stirred at 30 °C for 1 h and precipitated using an excess of ether. The precipitate was dialyzed against distilled water, and PLG was obtained after freeze‐drying (yield: 87%).

### Synthesis of PLG‐Fc‐III‐4C

PLG (11.3 mg, 0.34 µmol) and *N, N*‐disuccinimidyl carbonate (9.4 mg, 0.037 mmol) were dissolved in 3 mL of DMF under anhydrous and nitrogenous conditions, and 0.1 mL of trimethylamine was added. The solution was incubated at 37 °C for 72 h. Then, Fc‐III‐4C (10 mg, 0.0058 mmol) dissolved in 0.4 mL of DMF was added. After incubation at 37 °C for another 4 h, the solution was dialyzed against water and freeze‐dried.

### Preparation of PLG‐Fc‐III‐4C‐IgG

Both PLG‐Fc‐III‐4C and IgG were prepared as stock solutions at 1 mg mL^−1^ in PBS (0.01 m, pH 7.4). A mixture was then prepared by combining 993 µL of IgG with 7 µL of PLG‐Fc‐III‐4C to achieve a 13:1 mass ratio. The mixture was then incubated at 4 °C for 3 h with rotation to prepare PLG‐Fc‐III‐4C‐IgG, which was directly subjected to DLS and gel permeation chromatography (GPC) analysis after rotation.

### Preparation of PD1/CTLA‐4 BsAb, NP^aPD1^ and NP^aCTLA‐4^

PLG‐Fc‐III‐4C was prepared as a stock solution at a concentration of 1 mg mL^−1^ in PBS. For PD1/CTLA‐4 BsAb preparation, antibodies aPD1 and aCTLA‐4 were combined with PLG‐Fc‐III‐4C in PBS at a mass ratio of 9.75:3.25:1 and incubated at 4 °C for 3 h with continuous rotation, yielding a final antibody concentration of 2.4 mg mL^−1^. After incubation, the product was analyzed using DLS and GPC. PBS (0.01 m, pH 7.4) was used in all preparations.

For NP^aPD1^ preparation, aPD1 was mixed with PLG‐Fc‐III‐4C at a 13:1 mass ratio for 3 h at 4 °C, resulting in a final antibody concentration of 2.4 mg mL^−1^. Similarly, aCTLA‐4 was mixed with PLG‐Fc‐III‐4C under the same conditions for NP^aCTLA‐4^ preparation. For NP^aPD1^ + NP^aCTLA‐4^ administration, NP^aPD1^ and NP^aCTLA‐4^ were administered consecutively at a 3:1 mass ratio immediately after preparation.

FITC‐labeled aPD1 and PE‐labeled aCTLA‐4 were mixed with PLG‐Fc‐III‐4C and rotated at 4 °C for 6 h to verify whether PLG‐Fc‐III‐4C was attached to both aPD1 and aCTLA‐4 at the same time. Then, the fluorescent signals of BsAb were analyzed using flow cytometry.

### Characterizations

The chemical structures of PLG and PLG‐Fc‐III‐4C were analyzed using ^1^H nuclear magnetic resonance spectroscopy (^1^H NMR, Bruker, AV‐500) after dissolving in NaOH/D_2_O. The molecular weights of PLG, PLG‐Fc‐III‐4C, IgG, and PLG‐Fc‐III‐4C‐IgG were compared using GPC (Waters GPC 3 system, Waters Ultrahydrogel Linear Column, Waters, Milford, MA, USA). The specific conditions were as follows: polyethylene glycol with a narrow distribution was used as a standard, and PBS (0.2 m, pH 7.4) was used as the eluting solvent. The flow rate was 0.5 mL min^−1^, the temperature was 35 °C, and the sample concentration was 2 mg mL^−1^. All size measurements were performed with a Nano‐Particle Analyzer (Zetasizer Nano ZS, Malvern Instruments Ltd., Malvern, UK). TEM imaging was performed on 2% phosphotungstic acid on a carbon support film copper mesh using a Hitachi HT7800 transmission electron microscope (Tokyo, Japan) with an accelerating voltage of 100 kV. Flow cytometry (BD FACS Celesta Multicolor Flow Cytometer, Piscataway, NJ, USA) was used to detect the fluorescence of BsAb labeled with two fluorescent antibodies.

The molecular weight of aPD1, aCTLA‐4, and PD1/CTLA‐4 BsAb was determined via asymmetric flow field‐flow fractionation coupled with multiangle laser light scattering (AF4‐MALLS) on a Wyatt Eclipse DualTec system (Dernbach, Germany) with a DAWN HELEOS‐II (Santa Barbara, CA, USA) and an Optilab T‐Rex refractive index detector (Santa Barbara, CA, USA), both operated at a wavelength of 658 nm. The refractive index increment of the samples in the eluent was assumed to be dn / dc = 0.185 mL g^−1^. Wyatt Astra software was used for data acquisition and analysis.

### Stability

PLG‐Fc‐III‐4C‐IgG was prepared according to a previously described method using an IgG concentration of 1 mg mL^−1^ and PLG‐Fc‐III‐4C (13:1, w/w) and incubated in PBS (0.01 m, pH 7.4) at 4 °C for 3 h with rotation. The particle size of the PLG‐Fc‐III‐4C‐IgG was assessed by DLS for 7 consecutive days, from day 0 to day 6.

### Cells and Animals

CT26 and MC38 murine colon cancer cell lines were purchased from Shanghai Bogoo Biotechnology Co. Ltd. (Shanghai, China). Murine colon cancer cell lines CT26 and MC38 were purchased in Shanghai Bogoo Biotechnology Co. Ltd. (Shanghai, China). Briefly, the cells were cultured in RPMI 1640 medium containing high glucose, 10% FBS, 1% penicillin, and 1% streptomycin and incubated at 37 °C, 5% CO_2_, and 95% air (≈20% O_2_).

CD8^+^ T cells for the in vitro experiments were isolated from murine spleens. The CD8^+^ T cell isolation kit was purchased from StemCell (item No.: 19 858) and used according to the manufacturer's instructions. Spleens were carefully collected, washed three times with sterile PBS on ice, gently fragmented with the end of a syringe, and then suspended and filtered through a 250‐mesh nylon filter.

Female BALB/c and C57BL/6 mice were obtained from Beijing Vital River Laboratory Animal Technology Co., Ltd. (Beijing, China). All animal studies were conducted in accordance with the guidelines outlined in the Guide for the Care and Use of Laboratory Animals, and all procedures were approved by the Animal Welfare and Ethics Committee of Changchun Institute of Applied Chemistry, Chinese Academy of Sciences.

### In Vitro Immune Activation Capability of PD1/CTLA‐4 BsAb

Tumor cells were seeded in 96‐well plates at a density of 6 × 10^3^ cells per well, and cultured overnight. A total of 6 × 10^4^ CD8^+^ T cells were added to each well and co‐cultured with tumor cells in 150 µL of RPMI 1640 for 24 h. A total of 50 µL of fresh RPMI 1640 medium containing PBS, aPD1, aCTLA‐4, aPD1 + aCTLA‐4, NP^aPD1^ + NP^aCTLA‐4^, or PD1/CTLA‐4 BsAb with an equimolar of antibody was added into the wells. After treatment, the tumor cells were analyzed using the CCK‐8 assay, and perforin and granzyme B were analyzed in the supernatants by ELISA according to the manufacturer's instructions.

The concentration of aPD1 and aCTLA‐4 was 45 and 15 µg mL^−1^, respectively, in the MC38 and CD8^+^ T cell co‐culture experiment, and the drug treatment time was 48 h. For the CT26 and CD8^+^ T cell co‐culture experiment, the concentration of aPD1 or aCTLA‐4 was 90 or 30 µg mL^−1^, respectively, and the drug treatment time was 24 h.

### In Vivo Tumor Inhibition Experiment

The MC38 tumor model was established by the subcutaneous injection of 1.0 × 10^6^ MC38 cells into the right flank of C57BL/6 mice (female, 6 weeks old, 20 g). When the tumor volumes reached ≈50 mm^3^, the mice were randomly divided into six groups of six mice and treated with PBS (G1), aPD1 (G2), aCTLA‐4 (G3), aPD1 + aCTLA‐4 (G4), NP^aPD1^ + NP^aCTLA‐4^ (G5), or PD1/CTLA‐4 BsAb (G6). i.v. five times with 180 µg of aPD1 or/and 60 µg of aCTLA4 every other day. The day of treatment initiation was denoted as day 0.

The CT26 tumor model was established by the subcutaneous injection of 1.0 × 10^6^ CT26 cells into the right flank of C57BL/6 mice (female, 6 weeks old, 20 g). When the tumor volumes reached ≈50 mm^3^, the mice were randomly divided into six groups of six mice, and treated with PBS (G1), aPD1 (G2), aCTLA‐4 (G3), aPD1 + aCTLA‐4 (G4), NP^aPD1^ + NP^aCTLA‐4^ (G5), or PD1/CTLA‐4 BsAb (G6) by i.v. five times with 180 µg of aPD1 or/and 60 µg of aCTLA4 every other day. The day of treatment initiation was denoted as day 0.

Long and short tumor diameters were measured using a caliper, and body weight was measured and recorded every day. Tumor volumes were calculated using the formula: 1/2 × a × b^2^, where “a” is the long diameter of the tumor and “b” is the short diameter of the tumor. The TSR was calculated using the formula: TSR (%) = [(Vc – Vt)/Vc] × 100%, where “Vt” and “Vc” represent the mean tumor volumes of the treatment and PBS groups, respectively. Mice with tumors larger than 2000 mm^3^ were euthanized and considered dead.

### Biodistribution Assay

PLG (11.3 mg, 0.34 µmol) and *N, N*‐disuccinimidyl carbonate (9.4 mg, 0.037 mmol) were dissolved in 3 mL of DMF under anhydrous and nitrogenous conditions, and 0.1 mL trimethylamine was added. The solution was incubated at 37 °C for 72 h. Then, Fc‐III‐4C (10 mg, 0.0058 mmol) dissolved in 0.4 mL of DMF was added. After incubation at 37 °C for another 4 h, Cy5.5‐NH_2_ (9.000 mg, 0.013 mmol) was added and reacted at 37 °C for 24 h in the dark. The solution was dialyzed against water in a dialysis bag (MWCO = 7000 Da, Mengyimei Biotechnology Co., LTD, Beijing, China) and freeze‐dried. Cy5.5‐labeled PLG‐Fc‐III‐4C was obtained as a blue solid after freeze‐drying. PLG‐Fc‐III‐4C‐IgG/Cy5.5 and PD1/CTLA‐4 BsAb were prepared using the same method as described previously at a ratio 13:1 of IgG or mAbs to PLG‐Fc‐III‐4C/Cy5.5.

Murine colorectal MC38 tumor models were established by the subcutaneous injection of 1.0 × 10^6^ MC38 cells into the right flank of C57BL/6 mice (female, 6 weeks old, 20 g). When the tumor had reached ≈500 mm^3^, the mice were randomly allocated into three groups of three mice, and treated with PBS, PLG‐Fc‐III‐4C‐IgG/Cy5.5, or PD1/CTLA‐4 BsAb‐Cy5.5 by intravenous (i.v.) injection in 100 µL of PBS as the solvent. At 24 h after administration, the tumors and normal organs were harvested, and the biodistribution of the nanoparticles was imaged. The radiance was quantified using an IVIS Lumina LT Series III in vitro imaging system (PerkinElmer, Waltham, MA, USA).

Murine colorectal CT26 tumor models were established by the subcutaneous injection of 1 × 10^6^ CT26 cells into the right flank of BALB/c mice (female, 6 weeks old, ≈20 g). When the tumor volumes reached ≈500 mm^3^, the mice were randomly divided into three groups of three mice, and treated with PBS, PLG‐Fc‐III‐4C‐IgG/Cy5.5, or PD1/CTLA‐4 BsAb‐Cy5.5 (i.v.) in 100 µL of PBS as the solvent. At 24 h after administration, the tumor‐bearing mice were imaged, and the radiance was quantified using an IVIS Lumina LT Series III in vivo imaging system.

### In Vivo Toxicity

Healthy non‐tumor‐bearing BALB/c or C57BL/6 mice were treated with PBS, aPD1 + aCTLA‐4, NP^aPD1^ + NP^aCTLA‐4^, or PD1/CTLA‐4 BsAb (eq. to 180 µg/mouse aPD1, 60 µg/mouse aCTLA‐4) every 2 days by tail vein for a total of three doses. On day 5 post‐drug administration, the mice were euthanized, and the intestines were collected. H&E staining was used to observe the abundance of inflammatory cells in the mouse intestines.

### Immune Analysis

The proportions of tumor immune cells under different conditions were analyzed by flow cytometry. The day of the initial treatment was recorded as day 0. MC38‐bearing mice were euthanized on day 19 in the tumor inhibition experiment and on day 15 in the tumor inhibition experiment in CT26‐bearing mice. The tumors were harvested, gently ground, and digested in a solution containing 12 mg of collagenase, 1.2 mg of hyaluronidase, and 1.2 mg of deoxyribonuclease per 60 mL of 2% FBS supplemented RPMI 1640 medium and filtered through 250‐mesh nylon strainers to obtain single‐cell suspensions. The single‐cell suspensions were washed with PBS (0.01 m, pH 7.4) containing 2% FBS, stained with various fluorescent dyes conjugated to anti‐mouse antibodies for CD3, CD4, and CD8 and fixed with 4% paraformaldehyde. The levels of T lymphocytes (CD8^+^ T cells, CD4^+^ T cells, Treg cells) and natural killer (NK) cells in tumor tissues were assessed by flow cytometry (BD FACS).

### Cytokine Analysis

Peripheral blood was collected from mice after euthanization, allowed to stand for 30 min, and centrifuged at 1000 ×g for 15 min at 4 °C. The serum concentrations of IFN‐γ, TNF‐α, IL‐2, and IL‐12 cytokines were measured using an ELISA kit according to the manufacturer's instructions.

### H&E Staining

MC38‐bearing mice for the tumor inhibition experiment were euthanized on day 19, and CT26‐bearing mice for the tumor inhibition experiment were euthanized on day 15. Tumors were extracted, fixed overnight with 4% paraformaldehyde buffer, and paraffin‐embedded. They were cut into 5‐µm‐thick sections and stained with H&E. Histopathological changes were observed under a light microscope (Nikon TE2000U, Nikon Instruments Inc., Tokyo, Japan).

### Immunofluorescence Staining

MC38‐bearing mice for the tumor inhibition experiment were euthanized on day 19 and on day 15 for the tumor inhibition experiment in CT26‐bearing mice. The tumors were extracted, fixed overnight with 4% paraformaldehyde buffer, and paraffin‐embedded. The sections were deparaffinized, rehydrated, and incubated with citrate buffer for antigen retrieval. Then, the sections were immersed in 3% bovine serum albumin (BSA) and incubated to block endogenous peroxidase. Sections were incubated with rabbit anti‐mouse CD8 antibody (GB15068), followed by incubation with horseradish peroxidase (HRP)‐labeled goat anti‐rabbit IgG (GB23303), and immersed in CY3‐tyramide (G1223) solutions. Then, the tissues were microwaved to remove antibodies, incubated with rabbit anti‐mouse CD4 antibody (GB15064) and HRP‐labeled goat anti‐rabbit IgG (GB23303), and immersed in Alexa Fluor 488‐tyramide (G1231) solution. After counterstaining the nucleus with 4′,6‐diamidino‐2‐phenylindole (DAPI) and quenching the fluorescence, a slice scanner was used to detect and collect the images. Fluorescence was visualized by confocal microscopy (Zeiss, Oberkochen, Germany). All antibodies were from Servicebio (Wuhan, China).

### Statistical Analysis

All experiments were replicated at least three times. Data are expressed as means ± SD or means ± SEM and indicated in the figure captions. Multiple groups of experiments were statistically analyzed by one‐way ANOVA. The unpaired student's t‐test was used for the statistical analysis of two groups of experiments or annotated figures (“ns” indicates no statistical significance. *p < 0.05 was considered statistically significant, and **p < 0.01, ***p < 0.001 and ****p < 0.001 were considered increasingly significant).

## Conflict of Interest

The authors declare no conflict of interest.

## Supporting information



Supporting Information

## Data Availability

The data that support the findings of this study are available from the corresponding author upon reasonable request.
