# Supplementary material for: Polymeric Multivalent Fc Binding Peptides‐Fabricated Clinical Compounding Bispecific Antibody Potentiates Dual Immunotherapy Targeting PD1 and CTLA‐4
Source: Adv Sci (Weinh). 2024 Nov 28;12(3):2408899. doi: 10.1002/advs.202408899 (PMC11744713; doi:10.1002/advs.202408899)
Supplement: Supplementary file 1 — Supporting Information [file ADVS-12-2408899-s001.docx]

Supporting information

**Polymeric Multivalent Fc Binding Peptides-fabricated Clinical Compounding Bispecific Antibody Potentiates Dual Immunotherapy Targeting PD1 and CTLA-4**

*Zongyu Liu, Hongyu Chu, Weidong Zhao, Chenguang Yang, Tongjun Liu*, Na Shen*, Zhaohui Tang**


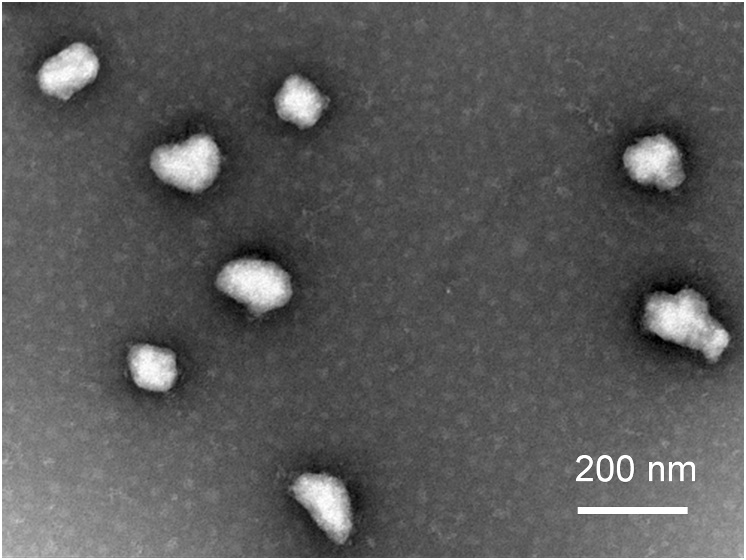


Figure S1. TEM image of PLG-Fc-III-4C. Scale bar, 200 μm.


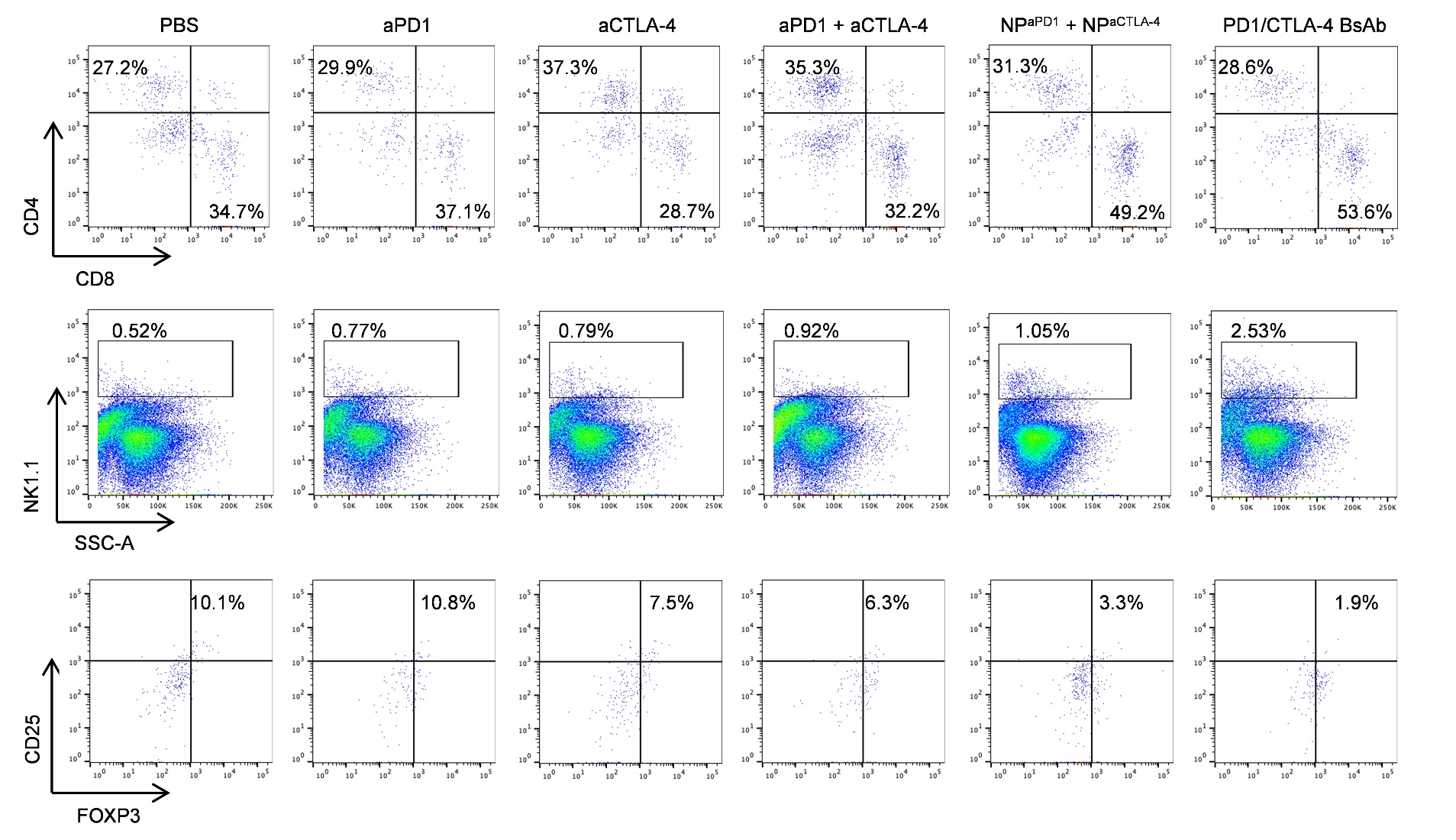


Figure S2. The effect of PD1/CTLA-4 BsAb on the proportion of different immune cells in tumors. Representative flow cytometric plots of CD8^+^ T cells, CD4+ T cells, NK cells, and Treg cells.


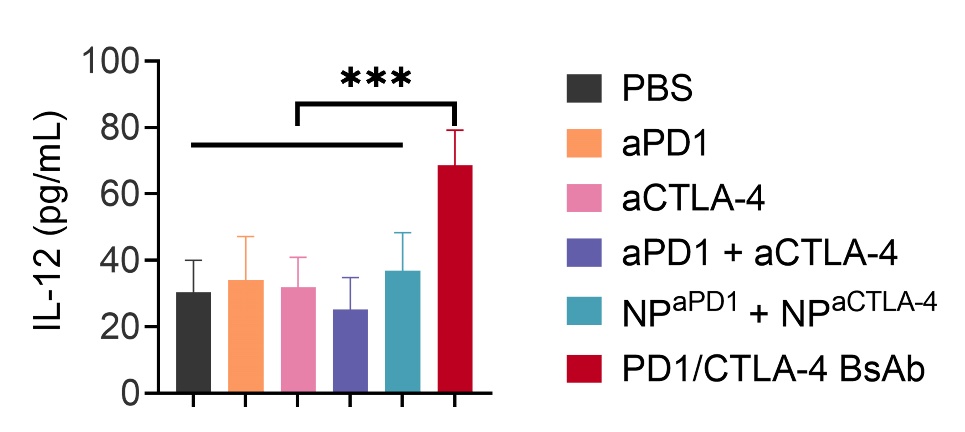


Figure S3. Serum IL-12 levels in MC38-bearing mice measured by ELISA.


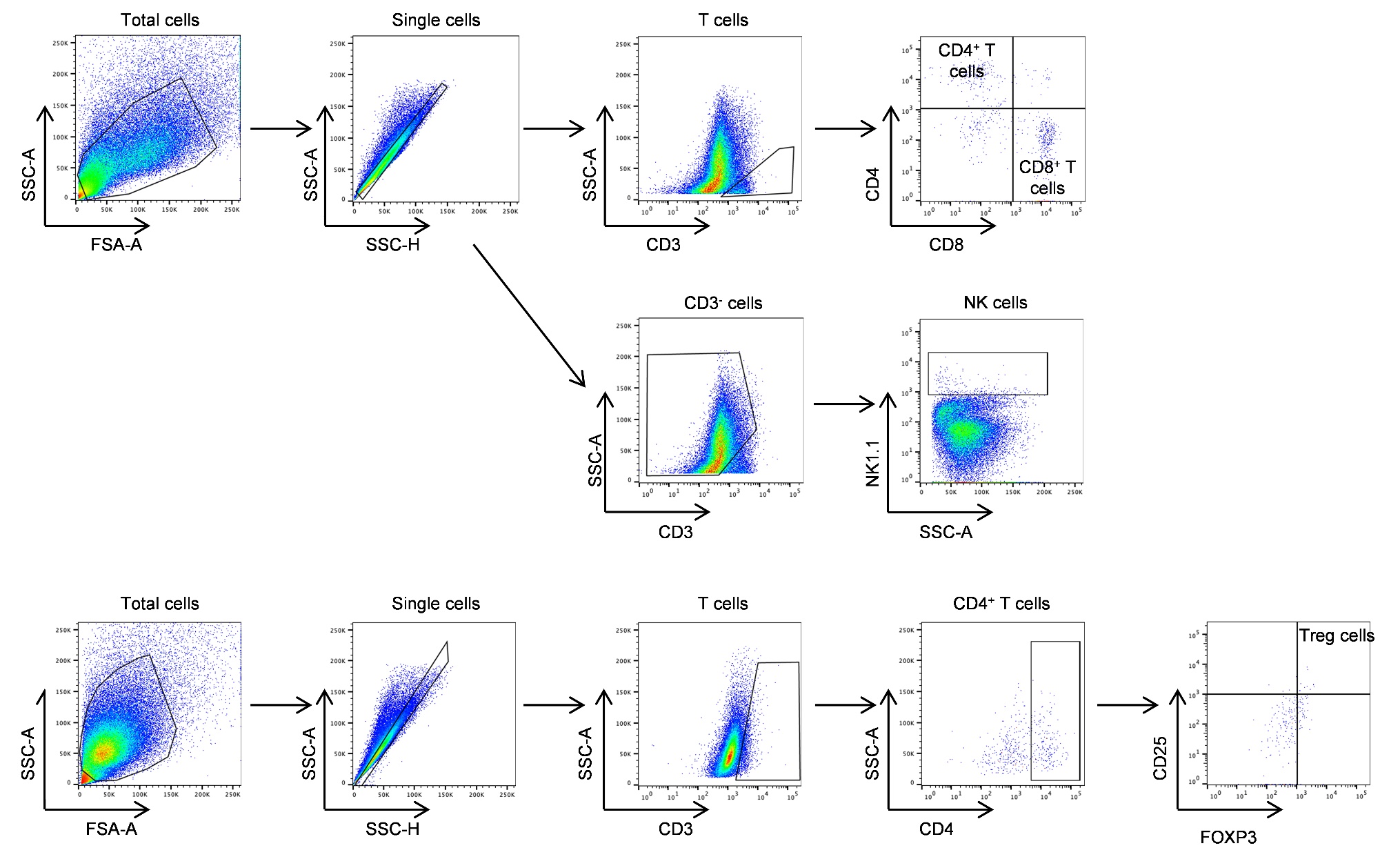


Figure S4. Gating strategy for flow cytometry to detect the intratumoral CD8^+^ T cells, CD4+ T cells, NK cells, and Treg cells in Figure 4.

## Table S1 Antibodies used in the experiment

| Antibodies | Company | Catalog | Application |
| --- | --- | --- | --- |
| FITC anti-mouse CD3 | Biolegend | 100203 | Flow cytometry |
| PE/Cy7 anti-mouse CD4 Antibody | Biolegend | 100422 | Flow cytometry |
| APC anti-mouse CD8a Antibody | Biolegend | 100712 | Flow cytometry |
| APC/Cyanine7 anti-mouse NK-1.1 Antibody | Biolegend | 156509 | Flow cytometry |
| APC anti-mouse CD25 Antibody | Biolegend | 101909 | Flow cytometry |
| PE anti-mouse FOXP3 Antibody | Biolegend | 126403 | Flow cytometry |
| FITC-labeled aPD1 | Biolegend | 135213 | Flow cytometry |
| PE-labeled aCTLA-4 | Biolegend | 106305 | Flow cytometry |
| anti-mouse PD1 antibody | Bio X Cell | BE0146 | Treatment |
| anti-mouse CTLA-4 antibody | Bio X Cell | BE0164 | Treatment |

Table S2 Data for calculating Cy5.5 loading content of PLG-Fc-III-4C/Cy5.5

| Standard curve of Cy5.5-NH_2_ | Concentration of PLG-Fc-III-4C/Cy5.5 | Absorbance at 681 nm of PLG-Fc-III-4C/Cy5.5 |
| --- | --- | --- |
| y=87.24*x+0.01242, R^2^=0.9983  (Note: y, Absorbance at 681 nm; x, mM) | 0.5 mg/mL | 0.9237 |
